# The VA’s Long-Term Care Strike Team Supporting Florida’s Nursing Homes Workforce

**DOI:** 10.1093/geroni/igab046.1476

**Published:** 2021-12-17

**Authors:** Adam Golden

**Affiliations:** Orlando VA Healthcare System, Orlando, Florida, United States

## Abstract

In coordination with the Florida Department of Health, the VA Sunshine Healthcare Network (VISN 8) established Long-Term Care Strike Teams to provide services to the LTC facilities most affected by the COVID-19 pandemic across the state of Florida. Between April 2020 through September 2020, the Strike Teams provided direct patient care to community residents, infection control/ prevention education, and patient/staff COVID-19 swabbing. We encountered facilities with large numbers of staff infected with COVID-19 and agency staff that were refusing to come to “COVID-infected” facilities. Remaining staff, including the administrators, were under much psychological distress. However, our experience supporting the long-term care facilities also had a major impact on our own perceptions of nursing home care. The bravery, dedication, and caring that we witnessed reinforced that the health care workers in long-term care facilities are true heroes.

